# Strengthening prevention of nutrition-related non-communicable diseases through sugar-sweetened beverages tax in Rwanda: a policy landscape analysis

**DOI:** 10.1080/16549716.2021.1883911

**Published:** 2021-04-20

**Authors:** Charles Mulindabigwi Ruhara, Safura Abdool Karim, Agnes Erzse, Anne-Marie Thow, Sylvere Ntirampeba, Karen J Hofman

**Affiliations:** a School of Economics, University of Rwanda, Butare, Rwanda; b SAMRC Centre for Health Economics and Decision Science Research-PRICELESS SA, University of the Witwatersrand, Faculty of Health Sciences, School of Public Health, Johannesburg, South Africa; c Menzies Centre for Health Policy, School of Public Health, Charles Perkins Centre (D17), The University of Sydney, Sydney, Australia; d Policy and Planning, Ministry of Youth and Culture, Kigali, Rwanda

**Keywords:** SSB taxation, NCD prevention, sugar-sweetened beverages, fiscal policies

## Abstract

**Background**: Food and beverages high in sugar are recognized to be among the major risk factors for nutrition-related non-communicable diseases. The growing presence of ultra-processed food producers has resulted in shifts to diets that are associated with non-communicable diseases and which include sugar-sweetened beverages. Sugar-sweetened beverage taxation presents an opportunity to prevent non-communicable diseases but it comes with challenges.

**Objectives**: To describe the policy landscape, identify and analyse the facilitators of and barriers to strengthening taxation on sugar-sweetened beverages in Rwanda.

**Methods**: We conducted a desk-based policy analysis to assess the facilitators of and barriers to strengthening sugary beverage taxation policy. We consulted eight stakeholders to validate the findings of the desk review.

**Results**: Non-communicable diseases are recognized as a public health challenge in Government health and non-health policy documents. However, sugar intake is not explicitly identified as a risk factor for non-communicable diseases and existing policies do not clearly aim to reduce sugar consumption. The Rwandan Government's commitment to growing the local sugar industry and the substantial economic contribution of Rwandan beverage producers are potential barriers to fiscal policies aimed at reducing sugar consumption. However, the current 39% excise tax levied on all soft drinks could support the adoption of future sugar-sweetened beverage policies.

**Conclusions**: The landscape for strengthening a sugar-sweetened beverage tax in Rwanda is complex. The policy environment provides both facilitators of and impediments to strengthening the existing tax. A differential tax could be introduced by leveraging on the existing excise tax and linking it to the sugar content of beverages.

## Background

The prevalence of nutrition-related non-communicable diseases (NR-NCDs) has increased rapidly worldwide [1]. Low- and middle-income countries (LMICs) have seen a surge in the prevalence of overweight and obesity, especially in urban settings, while, at the same time, not escaping the effects of undernutrition [2,3]. The major contributing risk factors are high-fat, high-sugar, energy-dense and micronutrient-poor diets [4]. In Rwanda, extensive health sector reforms have taken place over the past two decades and have brought improvements in public health indicators [5]. The country is one of the few African countries that achieved its Millennium Development Goal target for maternal mortality. It experienced a declining prevalence of communicable diseases, with the maternal mortality rate (MMR) declining from 1071 per 1000,000 live births in 2000 to 210 in 2015 [6].

However, the growing problem of non-communicable diseases (NCDs) has manifested as a major public health challenge in the country [7,8]. The most recent estimates by the World Health Organization (WHO) show that NCDs accounted for 36% of deaths in Rwanda [9]; cardiovascular disease, diabetes, chronic respiratory disease, cancers, injuries and disabilities are increasing [10,11]. For instance, in 2018, the cancer registry in Kigali City recorded 2,875 cases diagnosed and treated: 1167 males and 1708 females. The most common cancers for males were prostate cancer (17%), stomach cancer (10%) and lymphoma (8%), while those for females were breast cancer (21%), cervical cancer (20%), stomach cancer (8%) and lymphoma (5%) [12,13]. The impact on the health care sector is also notable: patients with chronic NCDs used 30–40% of adult hospitalization space in Rwanda [14].

Overweight, obesity and associated NR-NCDs are becoming increasingly prevalent in Rwanda. In 2014, 16% of women were overweight; 25% of women in urban areas and 30% in Kigali City were reported to be overweight, compared with 15% of women in rural areas [8]. Recent statistics showed that 6% and 10% of men were overweight in Rwanda overall and in the rural areas, respectively [8]. This increase in obesity is partly attributable to economic growth and urbanization over the last two decades, which have resulted in changing dietary habits for Rwandan consumers [9], specifically due to the change in their ability to purchase calorie-dense food products [13].

Much of this NCD burden is preventable by addressing the key risk factors of poor diet, tobacco use, alcohol consumption and physical inactivity [15]. Globally, sugar-sweetened beverage (SSB) consumption is strongly associated with weight gain and, consequently, NR-NCDs. Over-consumption of sugar is a key driver of obesity and diabetes, and SSBs contribute greatly to an individual’s sugar intake [16]. Research has established that regularly consuming 1–2 bottles of SSBs per day increases the risk of developing type 2 diabetes by 26% [17]. The rapid increase in consumption of SSBs has been identified as a major contributor to the rise of obesity and type-2 diabetes, amongst other diseases [17,18]. SSBs contribute a significant proportion of the daily energy consumption per person [19]. Specifically in Rwanda, the consumption of SSBs increased from 3,520 million RWF in 2005/2006 to 5,750 million RWF 2010/2011), and from the 18th to the 17th most consumed food product in urban Rwanda in those periods [20]. In addition to local consumption, Rwanda is also a main exporter of SSBs in the East African region [21].

Despite the policy efforts to address NCDs,  NR-NCDs have not been effectively addressed in Rwanda. Food policies are similar to those of other developing countries, and focus primarily on food production to address food insecurity, and under-nutrition to promote economic growth [22,23]. However, taxation on SSBs provides a policy mechanism to address this gap in LMICs. In particular, SSB taxation has emerged as a cost-effective strategy to address the increase in the prevalence of obesity and, thereby, reduce the incidence of NCDs [24,25]. Research has shown that taxing SSBs is associated with reduced purchases and consumption of those beverages, particularly among households of low socioeconomic status, due to increased prices [16,26].

However, studies have also highlighted the complex political economy of adopting these SSB taxes [24,27]. The process is often fraught with difficulties, and faces sophisticated opposition from private actors [25,28]. Despite the WHO’s recommendation of a 20% SSB tax [29] and growing evidence that taxation of SSBs has the potential to improve health outcomes [26], a tax aimed at reducing consumption of SSB has not yet been introduced in Rwanda. However, there is a revenue-generating excise tax of 39% on soft drinks, which applies to both SSBs and non-sugary beverages. Although the excise tax is a promising start, the inclusion of all soft drinks, irrespective of sugar content, has meant that it does not discriminate between SSBs and non-SSBs and is thus unlikely to cause a reduction in the consumption of SSBs. The strength of the existing excise tax means that there is a potential for the adoption of fiscal policies to reduce SSB consumption in Rwanda. There is a need to understand the policy context in Rwanda and the factors that may influence government’s ability to leverage on this existing tax for public health objectives.

The aim of the analysis reported in this paper was to describe the NCD policy landscape to identify and analyse the facilitators of and barriers to strengthening taxation on sugar-sweetened beverages in Rwanda. This study is one component of a larger regional study involving seven countries in sub-Saharan Africa [30].

## Methods

### Study design and frameworks

We conducted a desk-based qualitative policy analysis of documents related to NR-NCDs in Rwanda. We analysed the content of existing policies, investigated stakeholder attitudes towards SSB taxation, and assessed the broader political landscape. The methodology, with details about the study frameworks, is discussed elsewhere.

### Data collection

Publicly available Government policy documents (n = 9) and other relevant secondary data sources, including media reports, industry data and policymaker statements (n = 10) were identified through an online search. Details on the search strategy and inclusion criteria are presented in the study design paper [30].

We used the Walt & Gilson (1994) health policy analysis triangle, which focuses on policy context, content, actors and processes [31] to guide the systematic data extraction from policy documents into a matrix. We also extracted data regarding key stakeholders and their interests from the secondary data sources, drawing on Varvasovsky and Brugha’s (2000) approach to stakeholder analysis [32]. We obtained similar information on industry activities, using the framework developed by Mialon et al., 2015 [33] to identify and map corporate political activities of the beverage industry with respect to public health.

We consulted with eight stakeholders (policy-makers and academics) to verify the completeness and accuracy of our documentary data. These individuals were selected on the basis of their relevant expertise and professional responsibilities in relation to NCD prevention or SSB taxation policy. The consultations entailed i) confirming that all the relevant policy documents had been identified, and ii) requesting verification of our interpretation of the policy content and additional contextual data. No new data were collected during these consultations; the analysis relied exclusively on publicly available information obtained through the desk-based review. Consultations were conducted in English. Further detail on the approach to consultations is presented in the study design paper [30].

### Analysis

The lead author led the thematic analysis of policy content data, stakeholder data and data on corporate political activity with reference to Kingdon’s Framework for policy agenda setting, which focusses on policy change [34]. This framework describes three analytical ‘streams’, which we used to analyse our data. Our focus was on SSB taxation, with the objective of understanding the potential opportunities and barriers for policy change in this context, viz. problem stream, policy stream and politics stream. With respect to the problem stream, we examined the understanding of the problem of NR-NCDs relevant to SSB taxation. For the policy stream, we assessed the existing policy environment for NR-NCDs and SSB taxation. For the politics stream, we analysed the politics of key stakeholder groups as they related to NR-NCDs and SSB taxation.

## Results

The findings are summarised using Kingdon’s three streams [34] (Table 1). First, we describe the problem of NR-NCDs as framed within Rawandan Government policy documents. Second, we describe the content of key policies that address issues related to NR-NCDs. Third, we describe the political context around SSB taxation, including the positions of government, industry and other actors. For each stream, we discuss our findings and consider the facilitators of, and potential barriers to, the adoption of a SSB tax.

**Table 1.** Summary of the policy analysis findings using Kingdon’s three streams framework [34]

Stream	Topic	Factors facilitating SSB taxation	Barriers to SSB taxation
Problem	Current understanding of the problem of NR-NCDs	NR-NCDs considered a national problem, resulting in premature death & loss of productivity NR-NCDs impact on individual, family & community because of high cost of lifelong treatment & loss of productivity NR-NCDs recognised as an outcome of increased urbanisation	Primary focus of food policy is undernutrition and food security rather than unhealthy diet related to NR-NCDs Sugar not specified as a target risk factor for NR-NCDs – no explicit target for sugar consumption reduction
Policy	Existing policy environment addressing NCD intervention	Multi-sectoral approach to address NR-NCDs prevention Coherence between central and community-based interventions Excise tax of 39% levied on SSBs and non-SSBs; to increase Government revenue, not designed to support healthy consumption behaviour Beverage consumption considered to be elastic; sensitive to price in the last five years NR-NCDs interventions rely heavily on Government funding, which is considered to be unsustainable	SSB industry is one component of vision for national economic development Public–private partnerships exist to maximise value chain from growing, manufacturing and marketing of sugar-containing products, including SSBs Policy incoherence between Government’s vision for sugar industry and health goals to tackle NR-NCDs Access to quality drinking water, which is still a major challenge for large proportion of population, may encourage consumption of SSBs.
Politics	Stakeholder politics and SSB taxation	Opportunity exists for broad coalition of government and civil society players	Beverage industry is a duopoly, dominated by one large company – a subsidiary of a multi-national beverages concern Local industry positions itself as good corporate citizen by providing jobs, paying taxes, and investing in social development

### Stream 1: the current understanding of the problem of NR-NCDs (problem stream)

The policy documents in both the health and non-health sectors indicated that NR-NCDs are a recognized problem in Rwanda [7,35–37]. Overall, the problem of NR-NCDs is well-articulated in high-level policy documents. The 2018 Government National Strategy for Transformation (NST1) recognizes that NR-NCDs impact individual, family and community poverty because of the high cost of treatment and lifelong use of health services, as well as loss of productivity because of premature death at an economically productive age [35]. The Ministry of Health policies refer to medical evidence that NCDs were among the top 10 causes of mortality in district hospitals from as early as 2013 [7]. In fact, NR-NCDs accounted for at least 51.8% of all district hospital outpatients’ consultations and 22.3% of total district hospitalizations in that year [7]. Acknowledging the increasing national NCD burden as a significant public health threat, the Ministry of Health developed a dedicated NCD strategy which recognised the association of NCDs with increased urbanization and recommended the adoption of healthy lifestyles [37]. Key recommendations emanating from the 2013 NCDs risk factors report from the Ministry of Health included reduced cigarette consumption, and increased fruit and vegetable intake and physical activity [7].

Increased demand for processed and convenience food, related to urbanization, was identified as a driving cause of NCDs by the 2018 Agriculture Sector Strategic Plan, which promotes healthy food to curb NCDs [38].

Despite this, policy actions, to date, have focused on addressing undernutrition by increasing food production and the distribution of seeds, fertilizers and livestock to poor households, to increase food security through agricultural productivity [36]. Other forms of malnutrition, such as over-nutrition, have not been given adequate consideration, with implications for NCD policy formulation. We found no policy content that identified SSBs, let alone sugar, as a key NCD risk factor, even when these NCD prevention policies seek to address diabetes [37,39].

Thus, although the problem of NR-NCDs is recognized in Rwandan policies, consumption of sugar and SSBs is not identified as a key risk factor, and reducing consumption of sugar is not viewed as a measure to reduce the incidence of NCDs, i.e., policies do not explicitly discourage SSB consumption or sugar intake.

### Stream 2: existing policies for NR-NCDs prevention (policy stream)

Table 2 shows the range of government policy documents related to NR-NCDs that could support SSB taxation.

**Table 2.** Rwanda policy documents for NCDs

No.	Policy document	Content related to NCDs	Sectoral responsibility
1	National Transformation Strategy, 2017–2024 [35]	Highlights importance of NCDs and how they contribute to individual, family and community poverty because of high costs, including opportunity costs: treatment, lifelong medical care, and lost productivity from premature death in economically productive age groups.	Ministry of Health, with involvement of Education, Local Government, Natural Resources, Gender and Family Promotion, and Agriculture and Animal Resources Ministries
2	National Food and Nutrition Policy, 2014 [36]	Aims to prevent and manage all forms of malnutrition, increase knowledge about NR-NCDs, and develop and strengthen strategies to address NR-NCDs.	Coordinated by Ministry of Agriculture and Animal Resources
3	Rwanda Trade Sector for Quality, 2010 [45]	Ensures that goods and services, especially food emanating from or traded in Rwanda, are designed, manufactured and supplied in a manner that matches society’s needs and expectations, and those of regulatory authorities in local, regional and international markets.	Ministry of Trade and Industry responsible for overall coordination
4	Education Sector Policy, 2014 [41]	Tasks the education sector with developing curricula that address NCDs; emphasizes inter-sectoral cooperation.	Ministry of Education
5	Agriculture Sector Strategic Plan, 2018 − 2024 [38]	Strong focus on nutrition and food security, preventing NR-NCDs. Particular focus on infants and breast‐feeding mothers. Seeks to increase incomes and provide micronutrients to improve and prevent nutritional outcomes, including nutrient deficiencies and stunting in children.	Ministry of Agriculture and Animal Resources
6	Health Sector Policy, 2015 [51]	Promotes dietary diversity to address growing problem of over-nutrition. Recommends that dietary guidelines be developed with specific adaptations for groups and life stages with different nutrient requirements. Includes Government-approved standards for national mandatory fortification of industrially-milled wheat and maize flour, cooking oil, sugar and salt.	Ministry of Health
7	Non-Communicable Disease Policy, 2015 [39]	Makes several recommendations, including creating health- promoting environments; promoting community action to reduce exposure to modifiable NR-NCD risk factors and injuries; strengthening NR-NCDs prevention, diagnosis, care and treatment, and rehabilitation programs within the national health systems; and documenting national trends and determinants of NR-NCDs through monitoring and evaluation systems and research for evidence-based interventions.	Ministry of Health
8	Rwanda Non-Communicable Diseases National Strategic Plan, July 2014 – June 2019 [37]	Aims to reduce NCD-related morbidity and mortality in the Rwandan population. Target is to decrease mortality of under- 40s by 80% by 2020 and save around 8 300 lives per year. Outlines the following actions to achieve these targets: improved access and quality of care; improved general knowledge about prevention of risk factors and early detection; and development of a reliable M&E system, coordination and fund raising.	Ministry of Health
9	Fourth Health Sector Strategic Plan, July 2018 – June 2024 [11]	Outlines national strategic directions to improve health standards of Rwandans. Recommends improving coordination of stakeholders in nutrition program and production of healthy food crops, and increasing knowledge of good nutrition practices. Proposes to increase private sector engagement in nutrition and production of food commodities.	Ministry of Health in collaboration with social cluster Ministries

The Rwandan policy framework takes a multi-sectoral and community-based approach to NCD prevention. The nine documents outlined in Table 2 are telling examples of the cross-sectoral and collaborative nature of Rwanda’s policy response, which includes agriculture, health, trade, governance, education, and development processes. The NST1, for example, is focused on three areas: i) prevention through promotion of sports and early screening of chronic diseases; ii) scaling up specialized treatment options, including building capacity at different levels of the health care system and capacity of medical personnel; and iii) working with health insurance schemes to expand packages that are offered to NCD patients and that address issues of affordability [35]. The Rwanda NCD policy states that the prevention and control of NCDs should be integrated into existing health system structures from the central to the community level [39]. In line with the recommendation by the NST1 to strengthen disease awareness through community mobilization, regular free Government initiatives are undertaken for medical screening for hypertension and diabetes, and to calculate body mass index [36]. An annual one-week national sensitization campaign is designed to educate people about the dangers of tobacco, oil, beer and unhealthy foods, and the benefits of physical exercise [40]. The Ministries of Health and of Education collaborate to implement awareness-raising activities in schools to educate children and increase youth knowledge about healthy food and beverages, and the benefits of sport and exercise – instilling a culture of healthy dietary preferences among the younger generation [41]. Since 2013, at national level, planning for the NCD response has been coordinated by the NCD division of the Rwanda Biomedical Centre of the Ministry of Health [37]. Sectoral collaboration is essential because funding to address NCDs is shared across ministries and agencies [37].

The last decade has also seen the Rwandan government enact policies to strengthen its sugar industry. For example, in response to a climbing sugar price due to low production in 2011, the Rwandan Government introduced a 100% tax waiver on sugar imported from outside the country to stabilize the sugar price [42,43]. In 2015, in an effort to increase sugar production, a Dutch–Rwanda public–private partnership known as “*Sugar: Make it Work*” was established [44]. The main goal was to develop a more competitive, sustainable and inclusive sugar value chain in Rwanda, through addressing challenges of increasing demand as well as shortages of suitable land and irrigation, which hampered sugarcane growth, resulting in lower yields. There are other efforts from economic sectors, such as the ‘Made in Rwanda Agenda for the Sugar Industry’ policy, which aim to support and incentivise investment in a range of Rwandan industries, including the soft drinks industry [45]. The ‘Made in Rwanda’ policy and its Domestic Market Recapturing Strategy (DMRS 2015) prioritise the sugar, meat and pharmaceutical sectors for technological upgrading and a clear shift into higher value-added production, and are thus potential investment projects to be supported by the DMRS [45]. Currently, the country development employment and investment agendas for the sugar industry do not align with public health concerns about NR-NCDs. This is why strong actions related to cigarette consumption and physical exercise have been implemented by the Government of Rwanda, while no actions are specifically targeted against sugar intake.

In Rwanda, an excise tax of 39% is levied on soft drinks, including SSBs and non-SSBs, lemonades and other beverages, for revenue generation [46]. The tax is not implemented with the intention of reducing consumption of SSBs because it is applied to sugary- and other carbonated beverages, equally. Other beverages are also taxed, at varying rates, e.g. fruit juice at 5%, and brandy and whisky at 70%. The tax has not resulted in a significant price difference between SSBs and other beverages. For example, even with the excise tax, a 30 cl bottle of Coca Cola costs FRW 400 (USD 0.43), while mineral water of the same size costs FRW 300 (USD 0.32). Other factors related to drinking water, such as availability, distance to water sources, cost of collecting drinking water, and quality, may contribute to people’s preferences for SSBs. Only 57% of the population has access to safe drinking water within 30 minutes of their homes [47]. In rural settings, 55% of households take 30 minutes or more to collect drinking water [48].

Beverage consumption in Rwanda is, however, perceived to be elastic – an indication that an SSB tax could be effective [49]. The 2017 annual report of the dominant beverage manufacturer showed that, in the last 5 years, revenues were sensitive to price changes in soft drinks and beers, and declined from 2016 to 2017 [49].

The Rwanda policy environment shows how the health sector is funded. The health financing framework relies on two main channels for financing: 1) the supply side, which involves transfers from the treasury to health facilities and districts; and 2) the demand side, which comprises direct payment by households, together with the insurance system [50]. The Rwandan Government spends heavily on health but the current funding mechanisms are not adequate to address the problems associated with NCDs [50]. Government spending on health in the 2011/2012 fiscal year surpassed the 15% of Gross Domestic Product (GDP) required under the Abuja Declaration [51]. The direct payments by households, the insurance system, and the Community Based Health Insurance (CBHI) [50] are important components of health financing in Rwanda. The CBHI was designed to increase access, enhance affordability, and improve equitable financing of the health sector. Under the scheme, approximately 20% of the poorest citizens in the country have health insurance, from Government subsidies, of approximately USD 2.2 per individual per year. However, the total premium paid is less than the actual treatment costs, which include treatment for NCDs. Specific budget lines in the Ministry of Health exist to address nutrition and NCDs. Funds for NCDs have increased from less than 12 million USD in 2011 to around 15 million USD in 2014 [50]. Government spending provided more than 80% of the available funding for NCDs [50].

### Stream 3: stakeholder politics and SSB taxation (politics stream)

The overall NCD response is coordinated by the Ministry of Health but other ministries contribute, as specified in key policy documents [36–40,45,52]. Other important stakeholders include Government regulatory agencies (e.g. Rwanda Food and Drugs Authority), civil society organizations, consumer organizations, academia, research institutions, and media houses. Thus, the potential exists for a broad coalition to advocate for SSB taxation. However, little about these actors’ positions on SSBs or SSB taxation is publicly available, with most actors publicly indicating only a broad position in relation to NCD prevention.

The beverage industry in Rwanda is powerful. Documentary analysis showed that the industry is effective at constituency building, establishing relationships with opinion leaders and public health organizations, and promoting public–private interactions [49]. Two companies dominate the industry: Bralirwa Ltd with 80% of the market share, and Skol Ltd with 20% of the market share. Bralirwa Ltd produces and sells a portfolio of beer brands and soft drinks and has positioned itself as an important contributor to the economy [49]. It provides many jobs and is one of the largest taxpayers and employers in the country; and is a subsidiary of Heineken N.V. based in the Netherlands [49]. In addition, Bralirwa has a well-developed corporate social responsibility program, providing sponsorship for sports, music, arts development, and entertainment programs and events. Some of these programs are extensive, such as the solar-powered panel systems in Kayonza district [49]. Alongside Coca-Cola, Bralirwa funds projects to improve water, sanitation, and hygiene amenities for more than 50 000 people in Kicukiro district [49].

## Discussion

We analysed the Rwandan facilitators of and barriers to the adoption. Our findings show that the policy environment is broadly supportive, but competing priorities remain impediments to, the adoption of an SSB tax. Although the Rwandan Government has progressive cross-sectoral policies to address the growing burden of NCDs, these policies are in tension with policies that prioritise the growth of local sugar production and SSB industries. Though there is evidence and support for the use of fiscal policies to promote healthy dietary choices, competing priorities of economic growth in LMICs can be substantial barriers to their adoption and implementation.

Rwanda’s SSB taxation policies are distinct from those of many other countries. The existing excise tax of 39% on soft drinks is well above the recommended tax rate of 20% suggested by the WHO. However, unlike the SSB taxes adopted in South Africa or Mexico [27,28,52], this excise tax has not had a significant impact on the price or consumption of SSBs compared to non-sugary beverages. This is likely to be attributable to the structure of the tax, which applies equally to sugary- and non-sugary carbonates [46]. As a result, existing tax is unlikely to result in differential pricing or impact the consumption of SSBs. The tax also appears to have had a limited impact on the retail price of SSBs in comparison to other drinks, such as bottled water or fruit juices. This may be a result of industry lobbying for a favourable tax in support of the sale of SSBs, or the lower cost of producing SSBs as a result of tax exemptions and subsidies. In addition the government’s support for increasing local sugar production has led to policies which favour and support the manufacture of products like SSBs.

Nonetheless, the existing soft drinks excise tax may provide an entry point to the adoption of public health-oriented policies to reduce SSB consumption. If the excise tax was amended to target sugar content, it could change consumer behaviour and disincentivise SSB consumption, making healthier options more fiscally appealing to consumers [22]. Different taxes linked to the sugar content of beverages were adopted in countries like Mexico, the United Kingdom (UK) and South Africa. These resulted in increased prices of SSBs and also incentivised producers to reformulate their products to reduce the sugar content [52–54]. However, the adoption of these policies requires governments to shift from utilizing excise taxes solely to increase revenue, to including considerations of public health and health promotion in their fiscal policies.

An additional issue that policymakers must overcome is the perception of the economic impact of SSB taxes. For example, SSB taxes have been considered to be regressive for poorer consumers. Although this may be the case, this regressivity may be justifiable as, low-income households correspondingly experience the largest health benefits from taxes [16,55]. Other studies have established that high consumers of SSBs are likely to be price-responsive, which also increases the success of tax measures for health outcomes [56]. This is true in LMICs outside sub-Saharan Africa. For example, the specific tax of 10 pesos-per-litre for SSBs, introduced in Mexico, led to changes in purchasing behaviours and reduced consumption of SSBs [57].

As has been the case in many LMICs, the position and economic importance of the sugar and the SSB industry in Rwanda is likely to be a barrier to the adoption of SSB taxation. The Government of Rwanda is protective of its sugar industry and has taken several steps – including policy actions – to promote the supply of sugar and to assist farmers to compete in the regional sugar market. Current trade and agriculture policies are designed to strengthen the local sugar-producing industry by reducing imports and facilitating domestic production. Concerns about the economic and job implications of an SSB tax may also hinder or delay the adoption of such a policy. Our findings indicate that some stakeholders may be reluctant to implement an SSB tax due to the perception that it will negatively impact employment. Opponents to the adoption of an SSB tax in South Africa have argued that the tax would result in significant job losses, despite evidence to the contrary [28]. It may therefore be advisable for the Rwandan Government to limit the roles of private actors, such as the beverages industry, in the development phase of an SSB taxation policy.

The role of industry in SSB taxation policy may also be changing in Rwanda as a result of changes within the regional beverage market. Bralirwa’s large local presence is in line with a growing regional trend of local companies entering the bottled water, juice and SSB markets in their home countries [58]. These companies have expressed interest in expanding into neighbouring countries and markets. The East African Community (EAC) is a regional economic zone that can both facilitate the growth of the markets and African companies through the use of free trade mechanisms, and harmonize regulations such as the taxation of SSBs. The EAC secretariat has a technical working group on excise tax harmonization [58]. Should there be difficulties in adopting domestic interventions in Rwanda, interventions at a regional level may provide an alternative mechanism to improve NCD prevention efforts.

The policy landscape related to SSB taxation in Rwanda is influenced by a multiplicity of factors and is thus evolving. The existing policy landscape, at domestic and regional levels, provides a number of opportunities to strengthen SSB taxation but these are matched by a complex political landscape with competing priorities. Further action can be taken to improve support for an SSB tax and the successful adoption of a policy.

## Recommendations

Overall, our findings suggest that there are opportunities to strengthen the tax that currently applies to SSBs in Rwanda, which also align to the Government’s national development strategy, including both employment and public health goals. To be efficient, the tax rate should be linked to the sugar content with differential rates across categories of drinks. At the same time, efforts should be made to improve consumer access to alternative beverages, such as increasing the availability and quality of water. Such modifications would motivate the Government to utilize excise taxes to both increase revenue and promote health.

The role players that need to be engaged to facilitate such policy change include the Ministry of Health, Ministry of Finance and Economic Planning, Rwanda Revenue Authority, Rwanda Food and Drugs Authority, civil society organizations, consumer organizations, academia, research institutions, and media houses. The EAC should also be engaged so that it can propose adoption of regional regulations. The role of private actors, such as the beverages industry, in the development of NR-NCD policies should be limited.

Given the limited data available on SSBs and market drivers in Rwanda, there are many areas where further research is required. Individual knowledge, attitudes and behaviours associated with the consumption of SSBs are not informing Government NR-NCD policy documents. Given the Rwandan context, water access, quality and cost should also be documented to better understand how these factors may influence SSB consumption patterns in both urban and rural areas.

## Limitations

As a desk-based review, this analysis had several limitations. The primary limitation is that we relied on documents in the public domain. Nevetheless, through consultations, we ensured that the reviewed documents were considered by key stakeholders to be complete, up-to-date, and correctly interpreted. However, we encountered difficulties in obtaining publicly available information about industry stakeholders. Second, the unavailability of information about the beverage industry in Rwanda limited our ability to understand the role, activities, and influence of the industry within the policy landscape. Our insight into the broader political context of all stakeholders was limited to what was publicly available and accessible through a desk-based review. This resulted in a more detailed analysis of the policy and problem streams. Further research is needed to more fully understand the political context.

## Conclusion

There is an opportunity for the Rwandan Government to achieve both health and economic goals through reviewing the design of the current excise tax that includes SSBs, and through strategic stakeholder engagement and examination of the broader policy context. The 39% excise tax on carbonated beverages provides a good foundation for action. However, a structure that targets sugar content is needed for the tax to yield public health benefits. Having differential rates for SSBs, based on sugar content, could incentivize consumers to choose healthy alternatives, while simultaneously encouraging producers to reformulate their products. The broad NCD policy environment is supportive for action to prevent NCDs; however, current policies prioritize strengthening the sugar industry and may present a barrier to the adoption of a health-oriented SSB tax.

## Paper context

Sugar-sweetened beverages are key drivers of obesity and non-communicable diseases. We examined the Rwanda policy landscape for nutrition-related non-communicable diseases and analysed the facilitators of and barriers to the adoption of sugar-sweetened beverage taxation. There are tensions between public health goals, employment targets, and industry. In addition to Rwanda’s excise tax on sugar, there is a need for taxation of sugar content in beverages. The involvement of private actors in policy development should be limited.
